# CircSETD2 inhibits YAP1 by interaction with HuR during breast cancer progression

**DOI:** 10.1080/15384047.2023.2246205

**Published:** 2023-08-22

**Authors:** Lan Jing, Liu Yang, Cao Jianbo, Wan Yuqiu, Zhou Yehui

**Affiliations:** Department of General Surgery, The First Affiliated Hospital of Soochow University, Suzhou, People’s Republic of China

**Keywords:** CircSETD2, YAP1, HuR, breast cancer

## Abstract

CircRNAs have been proven to play a pivotal role in cancer progression. The present study aims to explore the roles and related mechanisms of circSETD2 in breast cancer proliferation, migration and invasion. The expression of circSETD2 in BC was assessed by the GEO database and qRT‒PCR. The biological function and underlying molecular mechanism of circSETD2 in BC were explored using in vitro and in vivo experiments, including CCK8, transwell, RIP, western blot, and xenograft mouse models. The expression of circSETD2 was downregulated in BC tumors, in accordance with the GEO database. Overexpression of circSETD2 significantly suppressed cell growth, cell migration and invasion. Mechanistically, circSETD2 reduced the stabilization of YAP1 by competitively binding with HuR, resulting in inactivation of downstream targets such as CTGF, myc and Slug. Our work suggests that the novel signaling axis circSETD2/HuR/YAP1 plays an important role in BC progression. The molecular mechanism underlying this signaling axis may provide a potential therapeutic target for BC treatment.

## Introduction

Breast cancer is a common disease that is harmful to women’s health. It has the highest incidence and the second highest mortality rate among female malignant tumor patients. On average, more than 1.3 million women worldwide are newly diagnosed with breast cancer each year, and more than half a million women die from it.^1,2^ At present, surgery, chemotherapy and radiotherapy are the basic methods of treatment for breast cancer.^3^ Although much research has been done on breast cancer diagnosis, the underlying molecular and cellular mechanisms remain to be explored.^4^ Therefore, the current focus is to elucidate the underlying mechanisms of breast cancer progression and metastasis and to find new targets for clinical treatment.

In recent years, research has confirmed that the progression and metastasis of breast cancer patients are regulated by genetics and epigenetics.^5^ In the study of epigenetics, noncoding RNA plays a significant regulatory role in tumor progression. CircRNAs are a new class of noncoding RNAs with a unique closed ring structure that are not easy to degrade and are more stable, so they are widely found in eukaryotic cells. Previous studies have indicated that circRNAs are involved in the occurrence and development of esophageal cancer, lung cancer, glioma, breast cancer and other tumors.^6–8^ Recent studies indicate that the abnormal expression of circRNA and the occurrence and development of breast cancer go hand in hand. For example, circTADA2As inhibit breast cancer progression and metastasis through targeted regulation of the miR-203A-3p/SOCS3 pathway.^9^ Another study showed that circRNA_103809 inhibits the proliferation and metastasis of breast cancer cells by regulating microRNA-532-3p.^10^ Although circRNA has been reported in breast cancer studies, the biological role of circRNA in breast cancer progression and metabolism has not been fully clarified.

By analyzing the GEO database, the results showed that circSETD2 was significantly reduced in BC tissues. Therefore, we chose circSETD2 to further analyze its role and mechanism in the progression of BC.

HuR, a member of the RNA-binding protein embryo lethal vision family, is also known as embryonic lethal vision and is widely expressed in mammalian cells. As an RNA-binding protein, HuR mainly regulates the expression of target genes through the posttranscriptional regulation mechanism of genes and then regulates tumor cell proliferation, differentiation, apoptosis, angiogenesis, lymphangiogenesis and other processes. HuR can be linked to a variety of regulatory factors, such as Von Hippel Lindau tumor suppressor (VHL), cyclin A, MMP-9, tumor necrosis factor (TNF), cyclooxygenase 2 (COX-2), VEGF, and p53.^11^ Previous studies have shown that HuR overexpression can be detected in ovarian cancer, gastric cancer, breast cancer, and cervical cancer tissues.^12–16^ Although there are many related studies on HuR, our understanding of its function and mechanism is still limited to a few cell types. In particular, little is known about the regulatory role of HuR and circRNA in breast cancer. Here, we focused on the biological roles of circRNA and HuR in BC and determined whether circRNA and HuR could affect tumor proliferation and invasion through their interactions.

In this study, we confirmed that circSETD2 is downregulated in breast cancer tissues and cells. In addition, circSETD2 suppressed cell migration, proliferation, epithelial-mesenchymal transition (EMT) and invasion via interaction with HuR and further downregulated YAP1. This study provides new insights into the regulatory mechanisms of circSETD2 and highlights a potential molecular target in BC. This study provides novel insight into the regulatory mechanisms of circSETD2 and highlights an underlying molecular target in breast carcinoma.

## Materials and methods

### Patients and specimens

A total of 30 cases were obtained from June 2018 to July 2021 in the First Affiliated Hospital of Soochow University (Soochow, Jiangsu Province, China). BC tissue samples and corresponding adjacent normal tissues were obtained from these patients.

After collection, these clinical tissue samples were stored at − 80°C until use. This work was approved by the Ethical Committee of the First Affiliated Hospital of Soochow University.

### RNA extraction and qRT‒PCR

Total RNA was prepared from tissues or cells using TRIzol reagent (Invitrogen, USA). To test the concentration and purity of RNA from cells or tissues, a spectrophotometer was used. Next, to synthesize cDNA, a PrimeScript RT reagent kit was used according to the manufacturer’s protocol. Then, following the procedure, we conducted quantitative real-time PCR by using the SYBR Green PCR Kit (Takara). GAPDH was used as an endogenous control.

### Cell culture and transfection

In this study, BC cell lines MCF7 and MDA-231 and human normal mammary epithelial cell line MCF-10A were purchased from American Type Culture Collection (ATCC, USA). The BC cell lines MCF7 and MDA-231 were cultured in Dulbecco’s modified Eagle’s medium (Corning, USA) with 10% FBS (Clark Bioscience). MCF-10A cells were cultured in Mammary Epithelial Basal Medium (MEBM, Cambrex, USA). All these cells were maintained at 37°C with 5% CO_2_. The full‐length circ SETD2 cDNA was inserted into the lentiviral vector pLCDH-ciR (Geenseed Biotech Co., Guangzhou, China) to achieve circSETD2 overexpression according to the manufacturer’s instructions.

### Cell proliferation assay

A CCK-8 assay was performed to test the effect of circSETD2 on the proliferation of cells. According to the manufacturer’s protocol, MCF7 or MDA-231 cells were seeded into a 96-well plate at a density of 1000 cells per well. After 24 h, 48 h and 72 h of cultivation, 10 μl of CCK-8 solution was added to the wells and incubated for 2 hours. Next, the optical density (OD) value of each well was measured at 450 nm by a microplate reader (Bio-Rad, Hercules, CA, USA).

### Transwell assay

Briefly, a 24-well-plate transwell chamber (Corning, NY, USA) precoated with Matrigel was used for the invasion assay, whereas a transwell chamber without Matrigel was used for the migration assay. The lower chamber of each well was filled with DMEM (0.5 ml) with 10% FBS. After 24 hours, the cells were fixed with paraformaldehyde for 40 min and

dyed with 0.1% crystal violet for 0.5 h. Finally, the cells were counted under a microscope.

### Xenograft animal studies

One-month-old female nude mice were purchased to perform xenograft animal studies. Approximately 1 × 10^6^ MCF7 cells were injected subcutaneously into the axilla of female nude mice. One month later, the mice were euthanized. The tumor was excised, photographed and measured.

### RNA immunoprecipitation (RIP) assay

The RIP assay was conducted with a Magna RIP™ RNA Binding Protein immunoprecipitation Kit (Millipore, Billerica, MA) in accordance with the manufacturer’s instructions. The extracted RNAs were determined by RT‒qPCR.

### Breast cancer RNA expression data retrieval and analysis

The RNA-Seq data of breast cancer were downloaded from the TCGA database (https://portal.gdc.cancer.gov). The circRNA expression profile data were downloaded from the GEO microarray database (GSE101123).

### Western blotting analysis

The total protein of MCF7 and MDA-231 cells was extracted with RIPA buffer, separated by SDS‒PAGE, and then transferred onto a PVDF membrane (Millipore, USA). Next, the membranes were blocked with 5% skim milk powder at room temperature for two hours. Thereafter, the membranes were incubated with primary antibodies overnight at 4°C, followed by HRP-labeled secondary antibody. Finally, the bands were examined by chemiluminescence.

### Statistical analysis

Statistical analyses were performed using SPSS 26.0 software and GraphPad Prism 7. Statistically significant differences were calculated using Student’s t test and expressed as a *P* value (*P < .05; **P < .01; ***P < .001; n.s. P > .05) as indicated in the individual figure legends.

## Results

### CircSETD2 was significantly downregulated during BC progression

**Figure 1.**
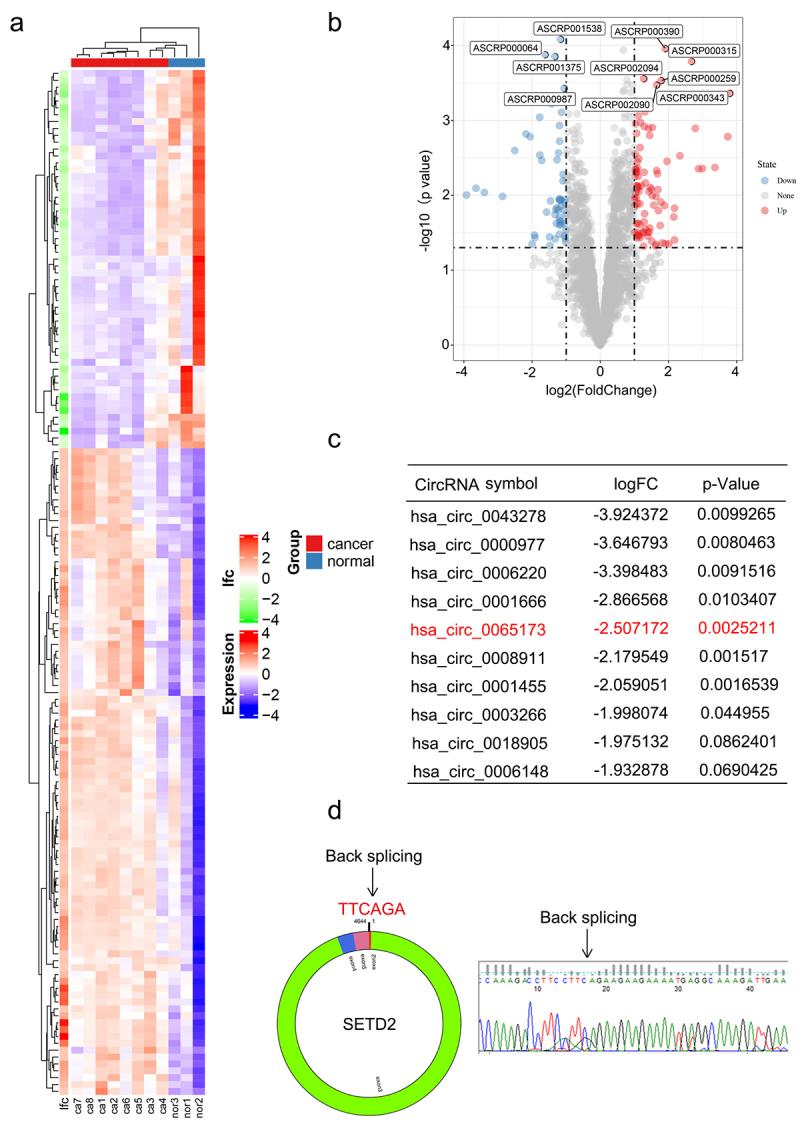
circSETD2 was downregulated in BC. (a-b) Heatmap and volcano map from the GEO microarray database (GSE101123) revealed the differentially expressed circRnas in BC and normal subjects (*P* value < 0.05 and log2-fold change > 1). (c) the top ten downregulated circRnas in GSE101123 are shown. (d) the diagram shows that circSETD2 is generated from exons 2–5 of the SETD2 gene. The back-splicing sites were validated by Sanger sequencing.

**Figure 2.**
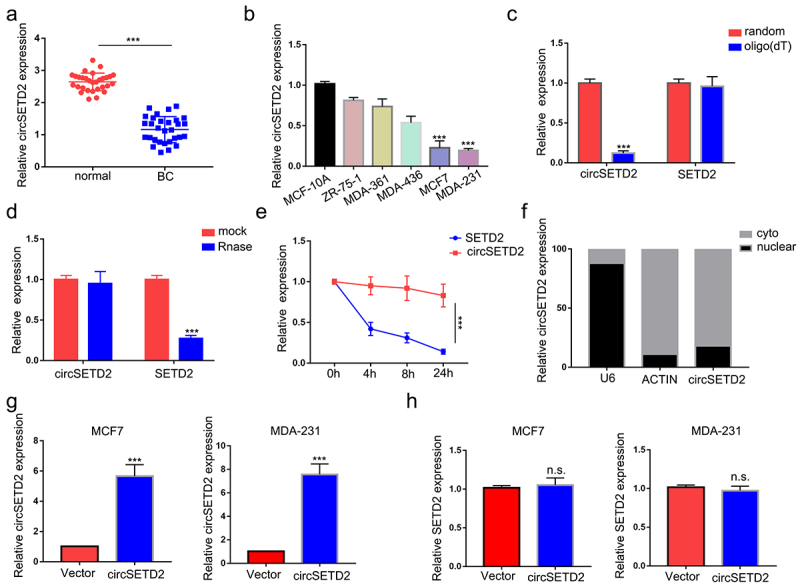
Characterization of circSETD2. (a) the expression of circSETD2 was measured by qRT‒PCR in clinical tissues. (b) the expression of circSETD2 was measured by qRT‒PCR in BC cell lines and nontumorigenic epithelial cell lines. (c) the levels of circSETD2 and linear SETD2 mRNA were detected after reverse transcription with random or oligo (dT)18 primers by qRT‒PCR. (d-e) after treatment with actinomycin D (d) and RNase R (e), circSETD2 and linear SETD2 mRNA expression levels were examined using qRT‒PCR in MCF7 cells. (f) the localization of circSETD2 was measured by subcellular fractionation. (g-h) the expression of circSETD2 and SETD2 was measured by qRT‒PCR after circSETD2 overexpression.

To identify differentially expressed circRNAs during BC progression, we first analyzed the microarray data GSE101123, which contained eight BC samples and three normal tissue samples, in the GEO database (Figure 1a–b). We identified a total of 208 distinct circRNAs with a 2-fold change (*p* < .05). Among them, 128 were upregulated and 80 were downregulated in BC samples compared with normal breast tissues. The top 10 downregulated circRNAs are shown in (Figure 1c). Since hsa_circ_0043278, hsa_circ_0000977, hsa_circ_0006220 and hsa_circ_0001666 have been reported and well elucidated in previous studies, they lack further research value. Hsa_circ_0065173, derived from the SETD2 gene exon 2 to exon 5, was also downregulated, and its role is still unknown in BC. Thus, we termed it circSETD2 and validated its sequence by Sanger sequencing (Figure 1d). Next, the characteristics of circSETD2 were elucidated by several experiments. Consistent with the microarray results, the expression of circSETD2 was downregulated in BC tissues compared with adjacent normal tissues (Figure 2a). For BC cell lines, circSETD2 was proven to be reduced in the BC cell lines MCF7 and MDA-231, particularly in contrast to that in the human mammary epithelial cell line MCF-10A (Figure 2b). To confirm the circularity of circSETD2, random hexamer and oligo (dT)18 primers were utilized. The reverse transcription efficiency of circSETD2 was significantly reduced by oligo dT primers due to the lack of a polyA tail (Figure 2c). Moreover, owing to the circular structure, endogenous circSETD2 was more stable than linear SETD2 mRNA with actinomycin D and RNase R treatment in BC cells (Figure 2d–e). To explore the localization of circSETD2 in BC cell lines, a subcellular fractionation assay and FISH assay were performed. As shown in Figure 2f and Figure S1A, circSETD2 mainly existed in the cytoplasm of BC cells. Collectively, these results suggested the circularity and characteristics of circSETD2 in BC tissues and cells.

### CircSETD2 inhibits the proliferation and metastasis of breast cancer cells in vitro and in vivo

**Figure 3.**
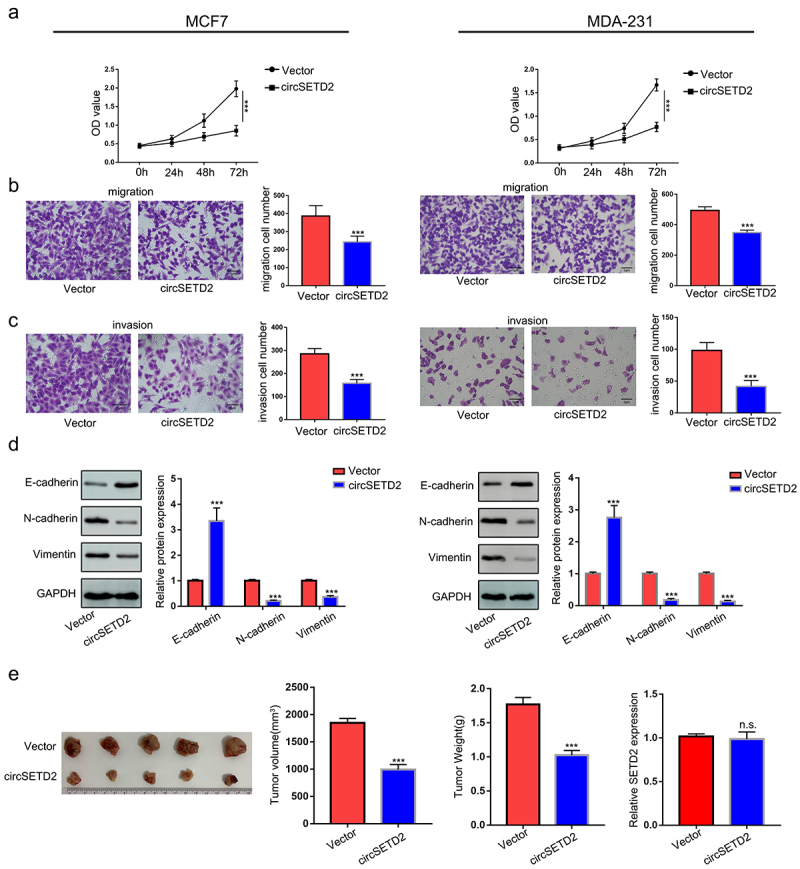
CircSETD2 suppressed BC cell proliferation, migration and invasion in vitro. (a) CCK-8 assay was used to measure the effect of circSETD2 on cell proliferation. (b-c) cell migration and invasion assays were used to measure the effect of circSETD2 on cell migration and invasion. (d) western blot assays were used to detect the expression of EMT markers. (e) a subcutaneous tumor formation experiment was used to measure the effect of circSETD2 on cell proliferation in vivo.

To explore the effect of circSETD2 on BC cell function, we constructed circSETD2-overexpressing MCF7 and MDA-231 cells (Figure 2g). The circSETD2 overexpression vector increased circSETD2 expression in both MCF7 and MDA-231 cells, while no significant change was found in SETD2 mRNA levels (Figure 2h). Next, several cell function assays were performed to detect the effect of circSETD2 on BC cell function. In the CCK-8 assay, overexpression of circSETD2 decreased the viability of both MCF7 and MDA-231 cells compared with those stably transfected with empty vector (Figure 3a). The results of cell migration and invasion assays showed that overexpression of circSETD2 significantly decreased cell migration and metastasis in both MCF7 and MDA-231 cells (Figure 3b–c). It is well known that epithelial-mesenchymal transition (EMT) plays a pivotal role during tumor metastasis and invasion processes. Next, we performed western blot analysis to examine EMT-related protein markers and evaluate the influence of circSETD2 on EMT. A rise in E-cadherin expression and a reduction in N-cadherin and Vimentin expression were observed in response to circSETD2 overexpression (Figure 3d). In summary, the above results demonstrate that circSETD2 suppresses the proliferation, migration, invasion and EMT progression of BC in vitro. Moreover, to further explore the effects of circSETD2 on BC cell proliferation in vivo, circSETD2-overexpressing MCF7 cells were subcutaneously injected into nude mice. As shown in (Figure 3e), both the volume and weight of xenograft tumors were significantly decreased in the circSETD2 overexpression group compared to the empty vector group. Accordingly, the qRT‒PCR assays also showed that no significant change was found in SETD2 mRNA levels in xenograft tumors. In summary, these results suggested that circSETD2 suppresses BC cell proliferation in vivo.

### CircSETD2 interacts with the HuR protein during BC progression

**Figure 4.**
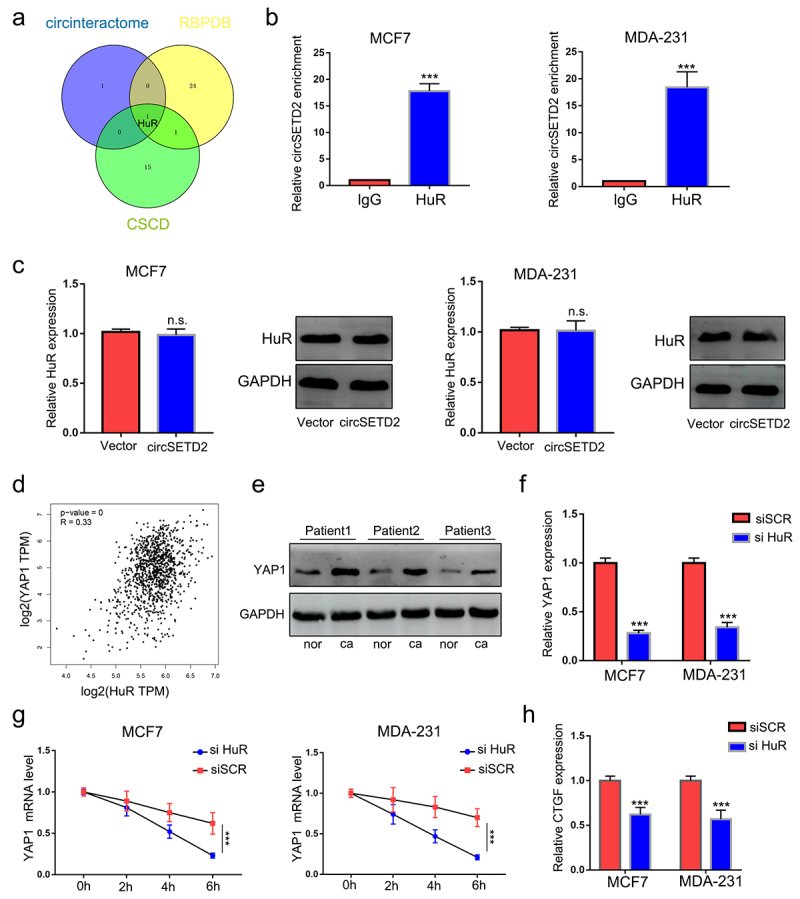
circSETD2 interacted with HuR. (a) circintractome, CSCD, and RPBDP databases were used to predict the potential proteins interacting with circSETD2. (b) the interaction between circSETD2 and HuR was confirmed by RIP assays. (c) qRT‒PCR and western blot assays were used to measure HuR expression. (d) the correlation between HuR and YAP1 was demonstrated by TCGA. (e) the expression of YAP1 in clinical tissues was detected by western blot. (f) the expression of YAP1 was measured by qRT‒PCR after HuR knockdown. (g) YAP1 mRNA levels were examined at different times after administration of actinomycin D. (h) the expression of CTGF was measured by qRT‒PCR after HuR knockdown.

To identify the potential protein partner of circSETD2, we performed bioinformatics analysis of circSETD2 by using several databases, including circinteractome, CSCD, and RBPDB. The results showed that only HuR was the most likely binding protein for circSETD2 (Figure 4a). To further validate the physical interaction between circSETD2 and HuR, RIP assays using anti-HuR antibodies were performed. As shown in (Figure 4b), circSETD2 was significantly enriched in the anti-HuR group compared with the lgG group, indicating that circSETD2 interacts with HuR in vivo. Moreover, overexpression of circSETD2 had no effect on HuR expression at either the mRNA or protein level (Figure 4c). Next, to explore the downstream molecular target of the circSETD2/HuR complex, bioinformatics methods (starBase, http://starbase.sysu.edu.cn/index.php) were used. In accordance with a previous study, the StarBase database predicted that HuR could potentially bind to YAP1, which has been proven to promote tumor progression.^17,18^ We also observed that YAP1 mRNA was positively correlated with HuR expression by analyzing breast cancer data from TCGA (Figure 4d). Western blot assays demonstrated that the protein level of YAP1 was also upregulated in BC tissues compared with normal tissues (Figure 4e). We next examined the RNA and protein levels of YAP1 in BC cells after HuR knockdown. The results showed that knockdown of HuR decreased YAP1 expression in both MCF and MDA-231 cells (Figure 4f; Figure S1B). Moreover, YAP1 mRNA stability was decreased in BC cells with HuR knockdown (Figure 4g). As expected, qRT‒PCR assays showed that knockdown of HuR also decreased the expression of CTGF, a target of YAP1 (Figure 4h).

### CircSETD2 suppresses YAP1 expression by reducing its mRNA stability

**Figure 5.**
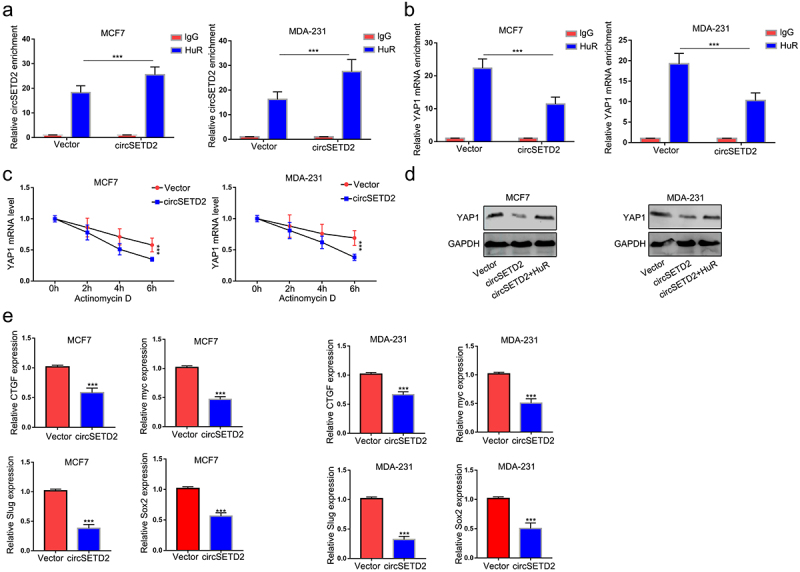
CircSETD2 suppresses YAP1 expression by reducing its mRNA stability. (a) the interaction between circSETD2 and HuR was measured by RIP-qPCR after circSETD2 overexpression. (b) the interaction between YAP1 and HuR was measured by RIP-qPCR after circSETD2 overexpression. (c) YAP1 mRNA levels were examined at different times after administration of actinomycin D in the circSETD2 overexpression and empty vector groups. (d) the expression of YAP1 was detected by western blot after circSETD2 and HuR overexpression. (e) the expression of CTGF, myc and Slug was measured by qRT‒PCR.

Previous results indicated that circSETD2 interacted with HuR and that HuR increased YAP1 mRNA stability. We wondered whether circSETD2 may impair the stabilization of YAP1 by competitively binding with HuR, leading to decreased transcription of downstream targets. To test this hypothesis, RIP assays were performed after circSETD2 overexpression. As shown in (Figure 5a–b), the interaction between HuR and YAP1 mRNA was significantly reduced in the circSETD2 overexpression group compared with the empty vector, whereas the interaction between HuR and circSETD2 was markedly increased after circSETD2 overexpression. Then, we tested the stability of YAP1 mRNA after circSETD2 overexpression. (Figure 5c) shows that the stability of YAP1 mRNA was reduced in the circSETD2 overexpression group in both MCF7 and MDA-231 cells. Western blot assays also demonstrated that overexpression of HuR rescued the inhibition of YAP1 expression by circSETD2 (Figure 5d). Moreover, the expression of YAP1 downstream targets, including CTGF, myc, SOX2 and Slug, was also reduced after circSETD2 overexpression (Figure 5e). Collectively, the above data elucidated that YAP1 is the downstream molecular target of the circSETD2-HuR axis.

### Overexpression of YAP1 rescues the inhibition of circSETD2 on BC cell metastasis progression

**Figure 6.**
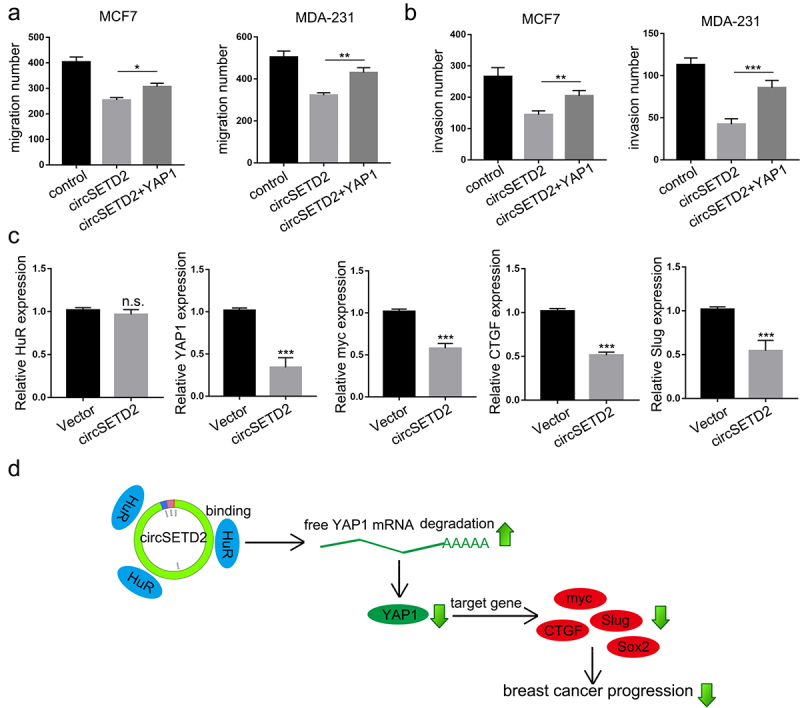
CircSETD2 suppressed BC progression via the HuR/YAP1 axis. (a-b) cell migration and invasion assays were used to measure the effect of circSETD2/YAP1 on cell migration and invasion. (c) the expression of HuR, YAP1 and downstream targets was measured by qRT‒PCR in xenograft tumors. (d) the schematic diagram shows that circSETD2 reduced the stabilization of YAP1 by competitively binding with HuR.

Next, transwell assays with or without Matrigel were used to investigate whether restoring YAP1 expression could attenuate the inhibition of circSETD2 in BC cells. The results demonstrated that the decrease in BC cell migration and invasion induced by circSETD2 overexpression was rescued by YAP1 overexpression (Figure 6a–b); Figure S1C-1D). Furthermore, as expected, the expression of YAP1 and its downstream targets myc, CTGF and Slug was also downregulated in circSETD2-overexpressing xenograft tumors (Figure 6c). Taken together, as shown in Figure 6d, our findings suggested that circSETD2 reduced the stabilization of YAP1 by competitively binding with HuR, resulting in inactivation of downstream targets such as CTGF, myc, SOX2 and Slug.

## Discussion

Breast cancer is the most common cancer in women worldwide and poses a serious threat to women’s health. Mounting evidence reveals that circRNAs play crucial regulatory roles in human tumorigenesis and development, suggesting that they may serve as prognostic biomarkers and therapeutic targets for cancer.^19^ Moreover, the GEO database was used to screen differentially expressed circRNAs in breast cancer tissues. We identified a novel circRNA, circSETD2. Hence, in the current research, we focused on the biological function and mechanism of circSETD2 in breast cancer. Our findings reveal that circSETD2 inhibits YAP1 by interacting with the HuR regulatory pathway in BC progression.

CircRNA, a stably expressed noncoding RNA, can not only act as a regulator of gene expression but also indirectly regulate the transcription of downstream genes by adsorption of miRNAs and regulate the expression of downstream genes by direct regulation of transcription and interference splicing. However, due to limited detection technology, single-stranded circRNAs are found in plant viruses. It is believed to be a missplicing phenomenon during exon transcription.^20^ Currently, circRNA has become a hotspot in biomedical research due to the rapid development of bioanalysis and high-throughput sequencing technology.^21^ An increasing number of studies have shown that abnormal circRNA expression is associated with the malignant behavior of a variety of cancers, including colorectal cancer, hepatocellular carcinoma, and lung cancer.^22^ Recent studies have also indicated that circRNAs can act as tumor drivers or tumor suppressors in a variety of ways.^23^ For instance, significantly upregulated circRNA_102231 expression in LAC tissue of lung cancer patients was associated with lymph node metastasis and poor overall survival.^24^ CircRNA_0000392 accelerates colorectal cancer progression by regulating miR-193A-5p expression and the PIK3R3/AKT pathway.^25^ In contrast, another study showed that hsa_circ_100395 restrains lung cancer progression through the miR-1228/TCF21 pathway.^26^ In our study, we found that the expression of circSETD2 in breast cancer tissues and cell lines was lower than that in adjacent normal tissues and human normal mammary epithelial cell lines. Hence, this result highlights the role of a novel circRNA in the regulation of tumor growth and invasiveness.

As an RBP, HuR is a key factor in posttranscriptional gene regulation and can achieve regulatory functions by affecting the translation, stability and cell localization of target mRNAs. Li et al. reported that HuR is involved in a large number of cellular events, such as proliferation, senescence, differentiation, apoptosis, stress and immune response.^27^ HuR is located mainly in the nucleus, and its cytoplasmic accumulation is the initial and key step of activation.^28^ Increased cytoplasmic expression of HuR inhibits the deadenylation and degradation process of ARE target genes, prolonging the life of the mRNA and resulting in abnormal protein increase. Numerous studies have shown that high cytoplasmic HuR expression correlates with the development of breast cancer, lung cancer, meningioma, or bladder cancer.^29^ For example, the expression of HuR in the cytoplasm was associated with HER2 negativity, PR and ER positivity, p53 expression, tumor grade and tubular differentiation phenotype, and it was found that the high expression of HuR in the cytoplasm could be used as a molecular marker for the diagnosis of invasive ductal breast cancer.^30^ Additionally, a previous study showed that HuR promotes the dryness of lung cancer cells by regulating the expression of miR-873 and miR-125a-3p.^31^ Nevertheless, the mechanisms that regulate these different effects of HuR in breast cancer remain unclear. Yan et al. indicated that miR-22 directly binds to the 3’-UTR of HuR, leading to the inhibition of HuR protein, further inhibiting the proliferation and migration of CRC in vitro and slowing the growth of CRC xenograft tumors in vivo.^32^ However, recent experimental results indicated that circRNAs could break the stabilization and translation of mRNAs by competitively binding with HuR.^33^ In this study, we found that overexpression of circSETD2 had no effect on HuR expression at either the mRNA or protein level. Consistently, we found that circSETD2 interacts with HuR, thereby regulating mRNA stability during BC progression.

YAP1 is known to be a transcription coactivator. Previous studies demonstrated that YAP1 is a vital oncogene in a significant number of cancer types.^34^ For instance, the YAP/TEAD complex, as a potential therapeutic target, can inhibit the effect of YAP1 deregulation on the progression of hepatocarcinoma.^35^ Another study showed that YAP1 can promote tumor angiogenesis and epithelial mesenchymal transformation by regulating miR-126-5p.^34^ Moreover, lncRNAB4GALt1-AS1 promotes osteosarcoma cell stemness and migration by recruiting HuR and enhancing YAP transcriptional activity.^36^ Meanwhile, in this study, the StarBase database predicted that HuR could potentially bind to YAP1. This will further support our hypothesis.

As a transcription coactivator, YAP1 can interact with transcription factors to activate downstream mRNA expression.^37^ In our study, our findings reveal greater expression of YAP1 protein levels in breast cancer tissues than in normal breast tissues. To explore the potential effect of YAP1, we knocked out the expression of HuR.

Intriguingly, we discovered not only downregulated YAP1 mRNA but also CTGF, the downstream target gene of YAP1. According to these results, we assumed that YAP1 is the downstream molecular target of the circSETD2-HuR axis. Taken together, we found that circSETD2 could competitively combine with HuR, therefore decreasing the stability of YAP1 and the expression of YAP1 mRNA and further inhibiting BC progression.

## Conclusion

In summary, this study uncovered the crucial role of circSETD2 in BC progression. We unveiled a new mechanism by which YAP1 regulates cancer proliferation and metastasis by interacting with HuR. Hence, this circRNA may be an underlying therapeutic target for breast carcinoma treatment.

## Supplementary Material

Supplemental Material

Supplemental Material

## Data Availability

The datasets used and/or analyzed during the current study are available from the corresponding author on reasonable request.
