# Supplementary figures and images for: CircSETD2 inhibits YAP1 by interaction with HuR during breast cancer progression
Source: Cancer Biol Ther. 2023 Aug 22;24(1):2246205. doi: 10.1080/15384047.2023.2246205 (PMC10446782; doi:10.1080/15384047.2023.2246205)

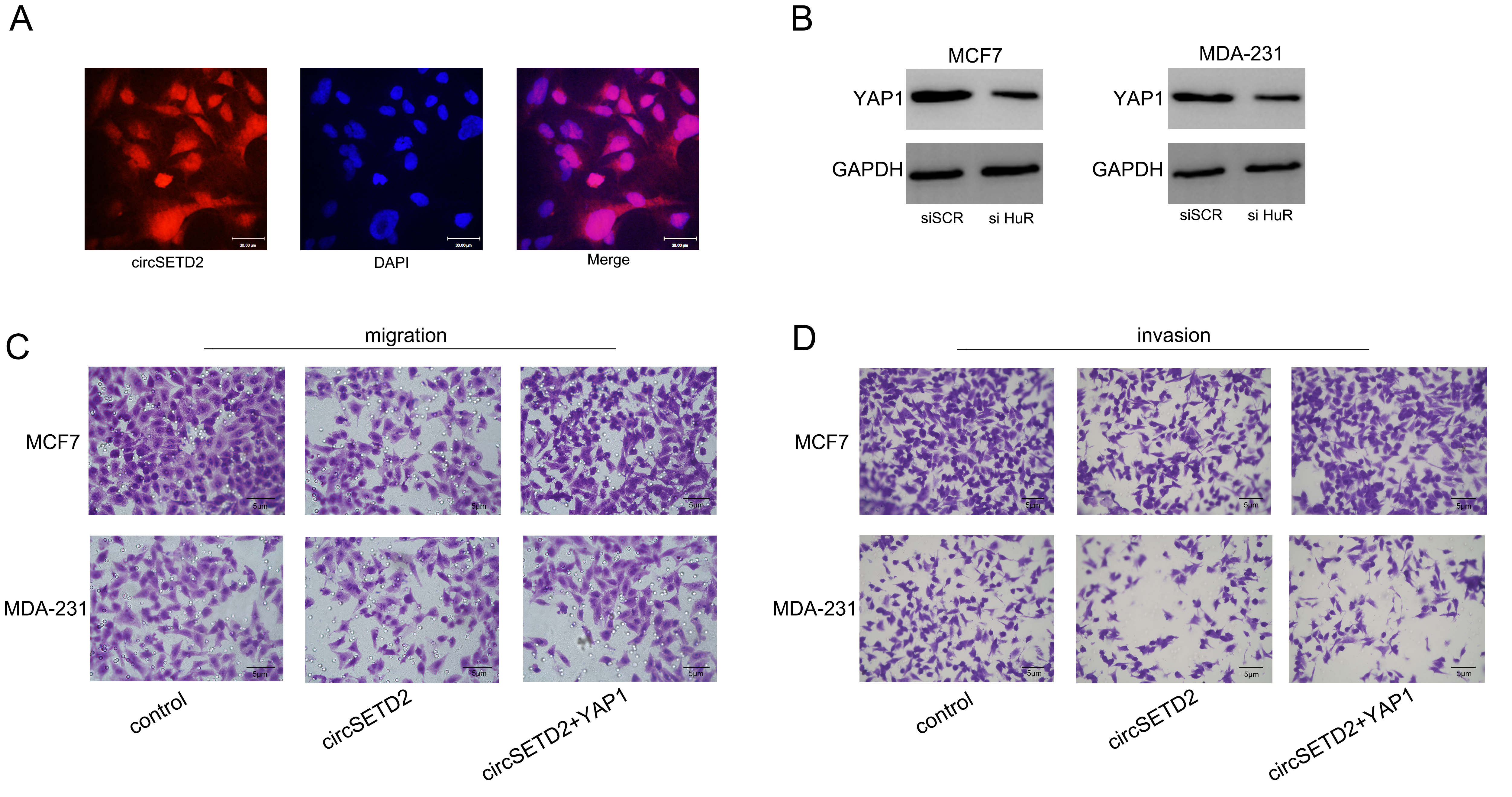

Supplement: Supplemental Material [file KCBT_A_2246205_SM0623.jpg]
