# Supplementary material for: CircSETD2 inhibits YAP1 by interaction with HuR during breast cancer progression
Source: Cancer Biol Ther. 2023 Aug 22;24(1):2246205. doi: 10.1080/15384047.2023.2246205 (PMC10446782; doi:10.1080/15384047.2023.2246205)
Supplement: Supplemental Material [file KCBT_A_2246205_SM0621.docx]

Figure S1. (A) The localization of circSETD2 was measured by FISH. (B) The protein level of YAP1 was measured by western blot assay after HuR knockdown. (C-D) Cell migration and invasion assays were used to measure the effect of circSETD2/YAP1 on cell migration and invasion.
